# Intention to Receive Cognitive Screening for Alzheimer’s Disease in Nondemented Older Adults

**DOI:** 10.1093/geroni/igaa057.838

**Published:** 2020-12-16

**Authors:** Juyoung Park, Magdalena Tolea, Lilah Besser, James Galvin

**Affiliations:** 1 Florida Atlantic University, Boca Raton, Florida, United States; 2 University of Miami Miller School of Medicine, Boca Raton, Florida, United States; 3 University of Miami Miller School of Medicine, Palm Beach Gardens, Florida, United States

## Abstract

The study explored factors associated with intention to receive cognitive screening for Alzheimer’s disease (AD). It also examined whether self-efficacy mediates the relationship between knowledge about screening and the intention to be screened. A population-based, random-digit dialing survey was performed; 1,043 responses were collected from a sample of nondemented older adults living in urban, suburban, and rural areas. A majority were female (66.8%, n = 697) and White (82.7%, n = 863) with a mean age 62.6 years (SD = 10.2). Findings from regression analysis identified that being female (β = .080), being depressed (β = .149), and having a positive life orientation (β = .120) were significantly associated with the intention to receive cognitive screening, p < .05. Results indicated that older adults with a positive life orientation reported greater intention to be screened for AD, whereas depressed participants were more likely to plan to be screened for AD. Bootstrapping results identified a mediating effect of self-efficacy (β = .2668, t = 7.3137, p < .0005). Self-efficacy mediated the relationship between knowledge about screening and intention to be screened. Using self-efficacy as a mediation effect indicated that older adults with knowledge about screening understand the benefits of early screening and diagnosis and are more likely to have self-efficacy (i.e., confidence to consult with a physician), and thus are more likely to show intention to be screened. Intention to be screened for AD could increase public awareness by defining effective ways to assist older adults to seek a cognitive screen.

